# PGE2 displays immunosuppressive effects during human active tuberculosis

**DOI:** 10.1038/s41598-021-92667-1

**Published:** 2021-06-30

**Authors:** Joaquín Miguel Pellegrini, Candela Martin, María Paula Morelli, Julieta Aylen Schander, Nancy Liliana Tateosian, Nicolás Oscar Amiano, Agustín Rolandelli, Domingo Juan Palmero, Alberto Levi, Lorena Ciallella, María Isabel Colombo, Verónica Edith García

**Affiliations:** 1 Departamento de Química Biológica, Facultad de Ciencias Exactas y Naturales , Universidad de Buenos Aires, Intendente Güiraldes 2160, Pabellón II, 4°piso, Ciudad Universitaria (C1428EGA), Buenos Aires, Argentina; 2 Instituto de Química Biológica de la Facultad de Ciencias Exactas y Naturales (IQUIBICEN), Facultad de Ciencias Exactas y Naturales, Universidad de Buenos Aires, Consejo Nacional de Investigaciones Científicas y Técnicas (CONICET) , Intendente Güiraldes 2160, Pabellón II, 4°piso, Ciudad Universitaria (C1428EGA), Buenos Aires, Argentina; 3 Laboratorio de Fisiopatología de La Preñez y El Parto, Centro de Estudios Farmacológicos Y Botánicos , CONICET-UBA, Buenos Aires, Argentina; 4 División Tisioneumonología, Hospital F.J. Muñiz, Uspallata 2272, (C1282AEN), Buenos Aires, Argentina; 5 Instituto de Histología y Embriología de Mendoza, Facultad de Ciencias Médicas, Universidad Nacional de Cuyo-CONICET, CP 5500 Mendoza, Argentina

**Keywords:** Tuberculosis, Antimicrobial responses

## Abstract

Prostaglandin E2 (PGE2), an active lipid compound derived from arachidonic acid, regulates different stages of the immune response of the host during several pathologies such as chronic infections or cancer. In fact, manipulation of PGE2 levels was proposed as an approach for countering the Type I IFN signature of tuberculosis (TB). However, very limited information regarding the PGE2 pathway in patients with active TB is currently available. In the present work, we demonstrated that PGE2 exerts a potent immunosuppressive action during the immune response of the human host against *Mycobacterium tuberculosis (Mtb)* infection. Actually, we showed that PGE2 significantly reduced the surface expression of several immunological receptors, the lymphoproliferation and the production of proinflammatory cytokines. In addition, PGE2 promoted autophagy in monocytes and neutrophils cultured with *Mtb* antigens. These results suggest that PGE2 might be attenuating the excessive inflammatory immune response caused by *Mtb*, emerging as an attractive therapeutic target. Taken together, our findings contribute to the knowledge of the role of PGE2 in the human host resistance to *Mtb* and highlight the potential of this lipid mediator as a tool to improve anti-TB treatment.

## Introduction

It is estimated that *Mycobacterium tuberculosis* (*Mtb*), the etiologic agent of tuberculosis (TB), has killed nearly 1000 million people in the last two centuries^1^. Furthermore, nowadays, TB remains a major global health problem ranking among the top ten causes of death worldwide. Despite the availability of an affordable and effective treatment, TB, alongside with COVID19 in 2020^2^, is the leading cause of death from a single infectious agent^3^. Moreover, there are nearly 2 million adult deaths annually from TB in developing countries, accounting for a quarter of the preventable mortality cases^3^. Thus, the main goals of developing new strategies against TB include reduction of treatment length, management of drug resistance problems, identification of safer treatments and eradication of drug interactions in patients with Human Immunodeficiency Virus (HIV) /TB infection^4^.

Although a vast progress has been made in characterizing the acquired immune response in TB patients, it remains to be elucidated the most appropriate defense mechanisms required to fight *Mtb*. The immune response against this pathogen is highly complex, and the outcome of the infection depends at least in part, on the role of several immune mediators with particular temporal dynamics on the host microenvironment. In this regard, in addition to the crucial function of cytokines and chemokines, accumulated evidence in recent years has indicated that different lipid mediators, mainly eicosanoids, are critical in the resolution of mycobacterial infection^5,6^.

Eicosanoids are a family of bioactive lipid mediators derived from arachidonic acid (AA), which is released from membrane phospholipids by phospholipases. There are three main pathways involved in the production of eicosanoids, which typically compete with each other for AA: (i) the cyclooxygenase pathway (COX-1 and COX-2) that produces prostaglandins and thromboxanes, (ii) the pathway of lipoxygenases (5-LO, 12-LO, and 15-LO), that catalyzes the production of leukotrienes and lipoxins, and (iii) the cytochrome p40 pathway, which generates hydroxyeicosatetraenoic and epoxieicosatrienoic acids^7^.

The ensuing findings that prostaglandin E (PGE) synthase-deficient mice^5^ and mice lacking the PGE2 receptor EP2 have increased susceptibility to *Mtb* infection^8^ provide strong evidence that the induction of apoptotic death of macrophages by PGE2 is critical for regulating *Mtb* growth in vivo^5^*,* although the exact mechanisms of PGE2 protection have not been elucidated. In this regard, Chen et al. reported that avirulent *Mtb* strain H37Ra induces PGE2 production, which protects against cell necrosis by preventing the internal mitochondrial membrane damage^5^ and promoting a rapid plasma membrane repair^9^. Conversely, high concentrations of PGE2 immunosuppress T cell-mediated immunity against *Mtb*^10^ and contributes to the expansion of regulatory T cells^11^*,* although the precise role of PGE2 in the development of adaptive immunity during human TB is uncertain.

Administration of both zileuton, a selective inhibitor of the enzyme 5-lipoxygenase (5-LO), and PGE2 to IL-1 signaling deficient mice or animals that induced excessive type I Interferon (IFN) showed host-beneficial effects^6^. Inhibition of 5-LO is proposed to shunt eicosanoid production toward increased PGE2 synthesis, which in the context of high-inflammatory environments suppresses detrimental type I IFN production, partially rescues mortality, and reduces bacterial burden^6^. Thus, manipulation of PGE2 and/or 5-LO could counteract these responses in patients with severe TB, opening new avenues to develop host-directed therapies (HDT). Hence, the functional characterization of soluble mediators that participate in the immune response against *Mtb*, would be crucial to improve vaccine designs and new immunotherapies that considerably improve control over TB. In fact, HDT impacting the eicosanoids production pathways have been previously proposed and some of them are already being tested at the clinical level (NCT02503839; NCT02781909; NCT04575519; NCT02060006).

Thus, considering that: (i) supplementing anti-TB therapy with host response modulators might augment standard TB treatment^12^; (ii) manipulation of the eicosanoid balance towards PGE2 can both prevent and therapeutically ameliorate disease exacerbation associated with type I IFN expression^6^; (iii) development of HDT in TB may be achieved by soluble mediators such as PGE2 that targets immune cells increasing their effector functions like autophagy^13–15^, and (iv) different clinical trials aim to inhibit the COX-2 enzyme as an HDT during TB treatment, we investigated the cellular and physiological bases of PGE2 function during human immune responses as a potential complement of anti-TB chemotherapy.

## Material and methods

### Subjects

Patients with TB (n = 40) were diagnosed at the Servicio de Tisioneumonología Hospital F.J. Muñiz, Buenos Aires, Argentina, based on clinical and radiological data, together with the identification of acid-fast bacilli in sputum. All participating patients had received less than one week of anti-TB regular therapy. Bacillus Calmette-Guerin (BCG)-vaccinated healthy control individuals (HD, n = 53) from the community participated in this study. Peripheral blood was collected in heparinized tubes from each participant after obtaining informed consent. The protocols conducted through the present work were approved by the Ethical Committee of Hospital F.J. Muñiz (ethical protocol number 1542/19). All methods were carried out in accordance with relevant guidelines and regulations.

### Inclusion and exclusion criteria

All subjects were 18–60 years old and had no history of illnesses that affect the immune system, such as HIV infection, a recent diagnosis of cancer, treatment with immunosuppressive drugs, hepatic or renal disease, pregnancy, or positive serology for other viral (e.g., hepatitis A, B or C), or bacterial infections (e.g., leprosy, syphilis). Individuals with bleeding disorders or under anticoagulant medication that might be at an increased risk of bleeding during the procedure of obtaining the sample were excluded from the study. Subjects with latent infection were excluded from the present study by using the QuantiFERON-TB^®^ GOLD PLUS (Qiagen, 622120 and 622526).

### Determination of plasmatic PGE2 levels

Plasma samples were collected from heparinized peripheral blood from HD and TB patients and centrifuged for 15 min at 1000×*g* at 4 °C. Plasma samples were then stored at − 70 °C. PGE2 measurement was performed by radioimmunoassay (RIA) as previously described^16^. Briefly, 50 µL of plasma were diluted with 50 µL of PGs RIA buffer (7.3 mM K_2_HPO_4_•3H_2_O, 2.7 mM KH_2_PO_4_, 145 mM NaCl, 7.1 mM BSA, 15.4 mM NaN_3_, pH = 7.4) and incubated with labeled (5,6,8,9,11,12,14,15(n)– ^3^H]–prostaglandin E2 (130 Ci/mmol, 100 μCi/mL, PerkinElmer, Buenos Aires, Argentina) for 1 h. Finally, a suspension of activated carbon (1%)-dextran (0.1%) was added to separate bound from free PGs. The samples were centrifuged at 2000*g* for 15 min at 4 °C and the supernatant was poured into vials containing 1 mL of scintillation liquid for aqueous sample. Radioactivity was measured in a beta scintillation counter. After a logarithmic transformation, the data were expressed as pg of PGE2/mL of plasma. The method has a cross-reactivity of less than 0.1% and a sensitivity of 5 pg/tube with a Ka = 1.5 × 10^10^ L/ mol.

### Antigen

In vitro stimulation of cells was performed with a cell lysate from the virulent *Mycobacterium tuberculosis* strain H37Rv, prepared by mechanic disruption (*Mtb*-Ag) (BEI Resources, NIAID, NIH: *Mycobacterium tuberculosis*, Strain H37Rv, whole cell lysate, NR-14822).

### Cell preparations and culture conditions

Peripheral blood mononuclear cells (PBMC) were isolated by centrifugation over Ficoll-Hypaque (GE Healthcare, 17-1440-03). Neutrophils were isolated from heparinized blood by centrifugation on Ficoll-Paque, dextran (Sigma, 31392) sedimentation, and hypotonic lysis^17^. Cells were suspended at 2 × 10^6^ in flat-bottom 24 or 48-well plates with RPMI 1640 (Invitrogen, 22400071) supplemented with L-Glutamine (2 mM, Sigma), Penicillin/Streptomycin, and 10% Fetal Bovine Serum (FBS; Gibco, 10,437,028) and cultured for 16 h without stimulus to allow monocyte adherence. Cells were then stimulated with lysated *Mtb* (*Mtb*-Ag, BEI Resources, NIH, 10 μg/mL) ± PGE2 (2 µM, Sigma, P0409) ± IFNα 2a (10 ng/mL, Biosidus) for different time points. In order to determine the effect of different treatments on the autophagic flux, the vacuolar-type HC-ATPase inhibitor Bafilomycin A1 (100 nM; Fermentek, 88,899–55-2) was added for the last 2 h of culture, before LC3 determination by flow cytometry. Viability was corroborated by staining with propidium iodide (PI; BD, 556547) and then analyzing by flow cytometry (Supplementary Fig.S1).

After isolation, neutrophil preparations were stained with an anti-CD14-PE (Biolegend, 325608) antibody and analyzed with a FACS Aria II cytometer (BD, San Jose, CA, USA) to guarantee that monocyte contamination was < 0.5% (Supplementary Fig. S2).

### Proliferation assay

PBMC were stimulated with *Mtb*-Ag for five days in the presence or absence of PGE2. Cells were pulsed with [^3^H]TdR (1 μCi/well, Perkin Elmer, MA, USA) and harvested 16 h later. [^3^H]TdR incorporation (c.p.m.) was measured in a liquid scintillation counter (Wallac 1214 Rackbeta, Turku, WF, Finland). Proliferation index for each individual was calculated as c.p.m. after *Mtb* Ag-stimulation/c.p.m. after culturing with medium.

### Flow cytometry

To determine the expression of immune receptors on lymphocytes, mononuclear phagocytes and neutrophils, cells stimulated with *Mtb*-Ag (10 μg/mL) treated or not with PGE2 (2 µM) were blocked in PBS (137 mM NaCl, 2.7 mM KCl, 8 mM Na_2_HPO_4_, and 2 mM KH_2_PO_4_)-FBS 5% for 15 min and then stained for surface expression with fluorophore-marked antibodies against SLAMF1 (BD, 559572), CD31 (BD, 560984), CD80 (Biolegend, 305207), Major Histocompatibility Complex (MHC) -I (Biolegend, HLA-A2, 343306), MHC-II (Biolegend, HLA-DQ, 318106), PD-L1 (eBioscience, MIH1), PD-L2 (BD, MIH18), CD3 (Biolegend, 300306) and CD14 (Biolegend, 367116).

Intracellular staining of endogenous saponin-resistant LC3 was performed as described^18^. Briefly, cells were washed with PBS and then permeabilized with PBS containing 0.05% saponin. In this protocol, the cells are not fixed, therefore LC3-I is washed out of the cell because, unlike LC3-II, it is not anchored to the autophagosome. Cells were then incubated with mouse anti-human LC3A, B antibody (MBL International, M152-3) for 20 min, rinsed with PBS, incubated with anti-mouse secondary antibody conjugated to fluorescein isothiocyanate (eBioscience,62-6511) for 20 min and rinsed twice with PBS. Afterwards, cells were stained with anti-CD14 or anti-CD3 antibodies (Biolegend, 325608 and 300308) to detect the monocyte and T lymphocyte populations.

Negative control samples were incubated with an irrelevant isotype-matched monoclonal antibody (Biolegend, 400140). Samples were analyzed on a FACSAria II flow cytometer (BD, San Jose, CA, USA). Supplementary Figure S3 shows the gating strategy for the analysis of flow cytometry experiments.

### ELISA

Culture supernatants of PBMC stimulated or not with *Mtb*-Ag in the presence or absence of PGE2 were obtained to evaluate cytokine levels by ELISA. TNFα and IFNγ secretion was measured by ELISA following the manufacturers’ instructions (BioLegend).

### ROS measurement

Neutrophils from HD and TB patients were incubated with 2ʹ,7ʹ-dichlorofluorescein diacetate (DCFDA, 50 µM; Invitrogen, D399) for 15 min at 37 °C. Then, cells were washed and stimulated with or without *Mtb*-Ag (10 µg/ml) ± PGE2 (2 µM) for 60 min. Finally, DCFDA fluorescence was evaluated to monitor ROS production by flow cytometry.

### Immunofluorescence microscopy

Cells were cultured and stimulated on coverslips for 16 h. After incubation under different experimental conditions, cells were washed in order to remove non-adherent cells. Adherent cells were then fixed with cold methanol for 20 s, then washed and subsequently permeabilized and blocked with blocking buffer (PBS containing 0.5% saponin; Santa Cruz Biotechnology, sc-280079A) and 1% bovine serum albumin (Santa Cruz Biotechnology, sc-2323A) for 15 min. The buffer was afterwards removed and the LC3 primary antibody was added (Cell Signaling Technology, 2775) and incubated for 16 h at 4 °C. Afterwards, cells were washed with blocking buffer and incubated with the secondary antibody (Alexa Fluor^®^ 488 Goat Anti-Rabbit IgG (HCL); Invitrogen, A11008) for 2 h at room temperature. Finally, nuclei were stained with DAPI. The coverslips were mounted with PBS-glycerol (Sigma-Aldrich, G2025) and fixed cells were imaged employing a Zeiss Spectral LSM 510 confocal microscope (Zeiss, Jena, Germany) using objective 63, numerical aperture (NA) 1.42.

Recombinant IFNɑ treatment efficiency was tested by NF-kB activation analysis. Briefly, PBMC from HD were stimulated with or without IFNɑ (10 ng/mL) for 30 min. Then, cells were stained with DAPI (Nuclei) and anti-NF-kB p65 antibody (Abcam, ab16502). Afterwards, NF-kB subcellular localization was examined by epifluorescence microscopy using an Olympus IX71 microscope (60X objective, NA = 1.25).

### Image processing

All the images were processed employing ImageJ software (Wayne Rasband, National Institutes of Health). After the image binarization using a defined threshold, the number of LC3 puncta was quantified utilizing the Particle Analyzer plugin. Brightness and contrast were adjusted in all images belonging to the same individual when needed.

### Statistical analysis

Analysis of variance and post hoc multiple comparisons tests were used as indicated in each figure legend. Mann–Whitney *U *test and Wilcoxon rank sum test were used for the analysis of unpaired and paired samples respectively. *P* values of < 0.05 were considered statistically significant.

## Results

### Study participants characteristics

Forty patients with active TB disease and 53 BCG-vaccinated, QuantiFERON^-^ healthy control individuals from the community were included. Demographic and clinical variables of the subjects included in the study are listed in Table 1.

**Table 1:** Demographic and clinical parameters of TB patients and healthy donors.

Characteristic	TB patients	Healthy donors
n	40	53
Age	26.3 ± 1.4	27.3 ± 1.9
**Sex (n = 40 and 53)**		
Male	16 (40%)	24 ﻿(45,3%)
Female	24 (60%)	29 ﻿(54,7%)
**Ethnicity (n = 40 and 53)**		
Caucasian (European lineages)	15 (37,50%)	32 ﻿(60,4%)
American Indian	24 (60%)	21 ﻿(39,6%)
Asian	1 (2,5%)	–
**AFB in sputum smear (n = 39)**		
AFB -	3 (7,69%)	–
AFB +	18 (46,15%)	–
AFB + +	10 (25,64%)	–
AFB + + +	8 (20,51%)	–
**Radiological lesions (n = 27)**		
Severe	13 (48,15%)	–
Moderate	11 (40,74%)	–
Mild	3 (11,11%)	–
Time of disease evolution (months)	2,21 ± 0,24	–
**Extra-pulmonary TB (n = 40)**		
Yes	2 (5%)	–
No	38 (95%)	–
**Immunological classification (n = 40)**		
High responders	14 (35,0%)	–
Low responders	15 (37,5%)	–
N/D	11 (27,5%)	–

Acid-Fast Bacilli (AFB) in sputum smear represent: AFB-, 0 bacilli count; AFB + , 1–9 bacilli/100 fields; AFB + + , 1–9 bacilli/10 fields; AFB + + + , 1–9 bacilli/field. Radiological lesions: "mild" corresponds to patients with a single lobe involved and without visible cavities; "moderate" relates to patients presenting unilateral involvement of two or more lobes with cavities, if present, reaching a total diameter no greater than 4 cm; "severe" corresponds to bilateral disease with massive affectation and multiple cavities. Clinical symptoms analyzed in TB patients previous to hospital admission to establish the time (months) of disease evolution were: weight loss, night sweats, symptoms of malaise or weakness, persistent fever, presence of cough, history of shortness of breath, and hemoptysis. Continuous data are expressed as mean ± SEM, and categorical data are expressed as number (percentages).

### TB patients display higher levels of plasmatic PGE2 than healthy donors

**Figure 1:**
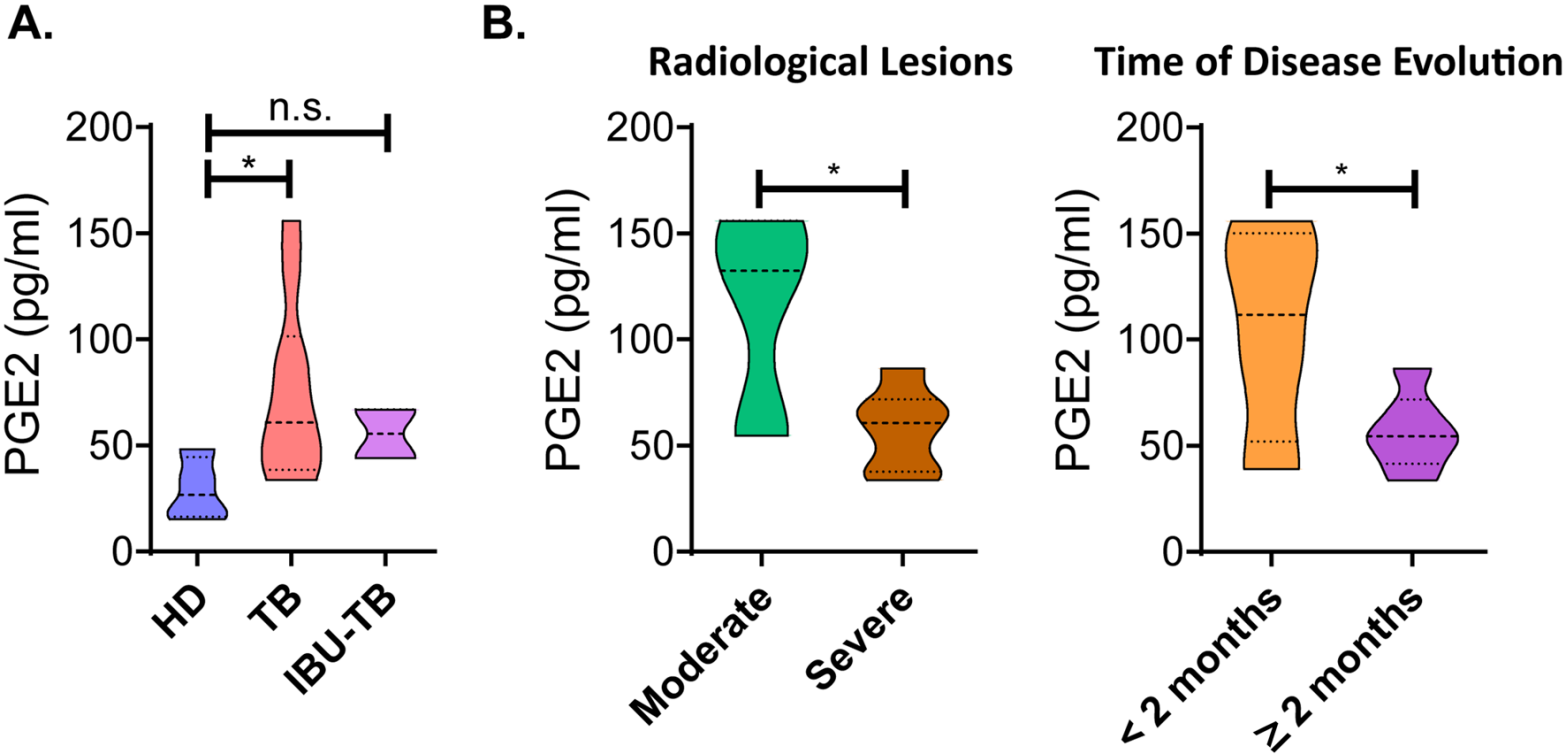
PGE2 plasma levels from Healthy Donors and tuberculosis patients. (**A**) Heparinized peripheral blood from HD (n = 5) and TB patients (n = 13) under ibuprofen treatment (IBU-TB) or not (TB) was centrifuged for 15 min at 1000 g and the levels of PGE2 in plasma were analyzed by RIA. (**B**) Plasmatic PGE2 levels in TB patients (n = 13) classified according to the severity of the disease. Briefly, the radiological lesions (moderate or severe) and the time of disease evolution (time previous to hospital admission during which the patient displays clinical symptoms) were determined. Violin plots show the median values of PGE2 plasma concentration (pg/ml) ± interquartile range. P values were calculated using the Mann Whitney non-parametric test for unpaired samples. * *p* < 0.05.

We initially determined PGE2 plasma levels in healthy donors (HD) and TB patients by RIA. In line with previous reports^6^, significantly higher concentrations of PGE2 were measured in patients with TB as compared to HD, which might reflect the inflammatory state of the infected individuals (Fig. 1). It is important to mention that a small percentage of patients (15%) were under treatment with ibuprofen at the recruitment time (400 mg every 6 h). As expected, due to the inhibitory function of ibuprofen on COX-2 enzyme activity, ibuprofen-treated TB patients displayed lower PGE2 levels as compared to non-treated TB patients.

We also analyzed the association between the PGE2 plasma levels in TB patients and the disease severity, as represented by the radiological lesions (moderate and severe) and the time of disease evolution (days previous to hospital admission during which the patient displays clinical symptoms). Thus, we observed that, in this cohort, patients with severe radiological lesions (bilateral and massive affectation with multiple cavities) exhibited lower amounts of PGE2 in plasma as compared to patients with moderate radiological lesions (Fig. 1B, left panel). Furthermore, we found lower amounts of plasmatic PGE2 in TB patients with a longer time of disease evolution in comparison with those that displayed a shorter time of disease evolution (Fig. 1B, right panel). No differences were observed regarding bacillary loads and neutrophil counts (Supplementary Fig. S4B, C).

In addition, we studied the relationship between PGE2 levels and the patient's immune state. Then, patients were classified according to our previously reported immunological classification^19^. Briefly, High Responder (HR) patients are individuals displaying significant proliferative responses, IFN-γ production, and an increased SLAMF1 expression against *Mtb*-Ag, whereas Low Responder (LR) patients exhibit low proliferative responses, IFN-γ secretion, and percentages of SLAMF1^+^ CD3^+^ lymphocytes. In the present work, we observed elevated plasmatic PGE2 levels in LR TB, patients with decreased T cell responses against *Mtb-Ag* in comparison with HR TB, individuals who display efficient anti-mycobacteria T cell responses (Supplementary Fig. S4A). Taken together, our findings highlight the relevance of the relationship of the clinical and immunologic state of TB patients with their levels of PGE2 in plasma.

### PGE2 regulates the expression of surface receptors on immune cells from HD and TB patients

**Figure 2:**
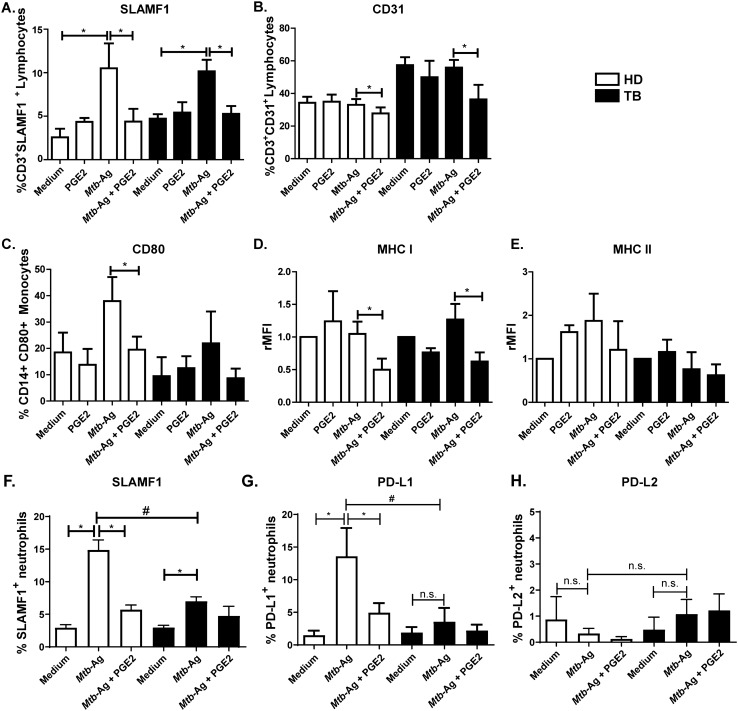
PGE2 regulates the expression of surface receptors on PBMC and neutrophils from HD and TB patients. (**A**–**E**) PBMC from HD (n = 7) and TB patients (n = 11) were stimulated with *Mtb*-Ag (10 µg/ml) in the presence or absence of PGE2 (2 µM) for 5 days. Afterwards, the surface expression of (**A**) SLAMF1 and (**B**) CD31 on CD3^+^ T cells and (**C**) CD80, (**D**) MHC-I and (**E**) MHC-II on CD14^+^ mononuclear phagocytes were determined by flow cytometry. Each bar represents the mean percentage of (**A**–**C**) CD3^+^ or (**D,E**) CD14^+^ Receptor^+^ cells ± SEM or relative Mean Fluorescence Intensity (rMFI) ± SEM. (**F**–**H**) Human purified neutrophils from HD (n = 7) and TB patients (n = 6) were stimulated with *Mtb*-Ag (10 µg/ml) in the presence or absence of PGE2 (2 µM) for 2 h. Then, (**F**) SLAMF1, (**G**) PD-L1 and (**H**) PD-L2 expressions were evaluated by flow cytometry. Bars represent the mean values of the percentage of Receptor^+^ neutrophils ± SEM. Statistical differences were calculated using one-way ANOVA and post hoc Dunnett’s multiple comparison test. * *p* < 0.05. # *p* < 0.05; Mann–Whitney nonparametric test for unpaired samples.

Subsequently, we decided to evaluate the effect of PGE2 on the expression of surface receptors involved in T lymphocyte activation. We previously reported that SLAMF1, a receptor that influences cytokine production by activated T cells, enhanced IFN-γ secretion against *Mtb*^20,21^, whereas the association of SLAMF1 with the adaptor SLAM-associated protein (SAP) inhibited IFN-γ production in patients with TB^19,22^. Furthermore, we also demonstrated that CD31 binds to SAP^23^. Thus, considering that both SLAMF1 and CD31 receptors have key regulatory roles in the signaling pathway(s) leading to the IFN-γ response against *Mtb* we aimed to investigate the modulation of PGE2 on the expression of these proteins. Thus, PBMC from both HD and TB patients were stimulated with lysate *Mtb* (*Mtb*-Ag) in the presence or absence of PGE2 for 5 days. Interestingly, we observed that PGE2 treatment significantly decreased SLAMF1 and CD31 expression on CD3^+^ T lymphocytes´ surface (Fig. 2A,B and Supplementary Fig. S5). Moreover, a marked reduction in the expression of the costimulatory molecule CD80 and MHC class I was also confirmed on CD14^+^ mononuclear phagocytes treated with this eicosanoid (Fig. 2C,D and Supplementary Fig. S6). Besides, we also detected a slight trend to a decrease in MHC class II expression on HD’s CD14^+^ cells stimulated with *Mtb*-Ag in the presence of PGE2 (Fig. 2E).

A direct correlation between the number of neutrophils and TB severity disease has been previously described^24,25^. Moreover, a duality between the ability of neutrophils to clear the infection and the contribution of high numbers of these cells to inflammation, disease severity and mortality has been proposed^24^. Thus, we then investigated the effect of PGE2 treatment on the expression of surface receptors on neutrophils from HD and TB patients. Then, human purified neutrophils from HD and TB patients were stimulated with *Mtb*-Ag in the presence or absence of PGE2. After 2 h, SLAMF1, PD-L1 and PD-L2 expressions were evaluated by flow cytometry. As shown in Fig. 2F, *Mtb*-Ag stimulation increased the surface expression of SLAMF1 on neutrophils from HD and TB patients as we previously reported^16^. In contrast, antigen stimulation augmented PD-L1 levels only on neutrophils from HD (Fig. 2G). Interestingly, treatment with PGE2 significantly reduced SLAMF1 and PD-L1 expression on neutrophils membrane (Fig. 2F,G Supplementary Fig. S7). Moreover, PD-L2 levels were not modulated by *Mtb*-Ag (Fig. 2H). Of note, we observed that neutrophils from HD downregulated CD11b expression after PGE2 treatment (Supplementary Fig. S8).

Therefore, our findings highlight an immunosuppressive role of PGE2 by decreasing the levels of several receptors on *Mtb*-Ag stimulated immune cells from TB patients and HD.

### PGE2 displays immunosuppressive effects on human PBMC and neutrophils

**Figure 3:**
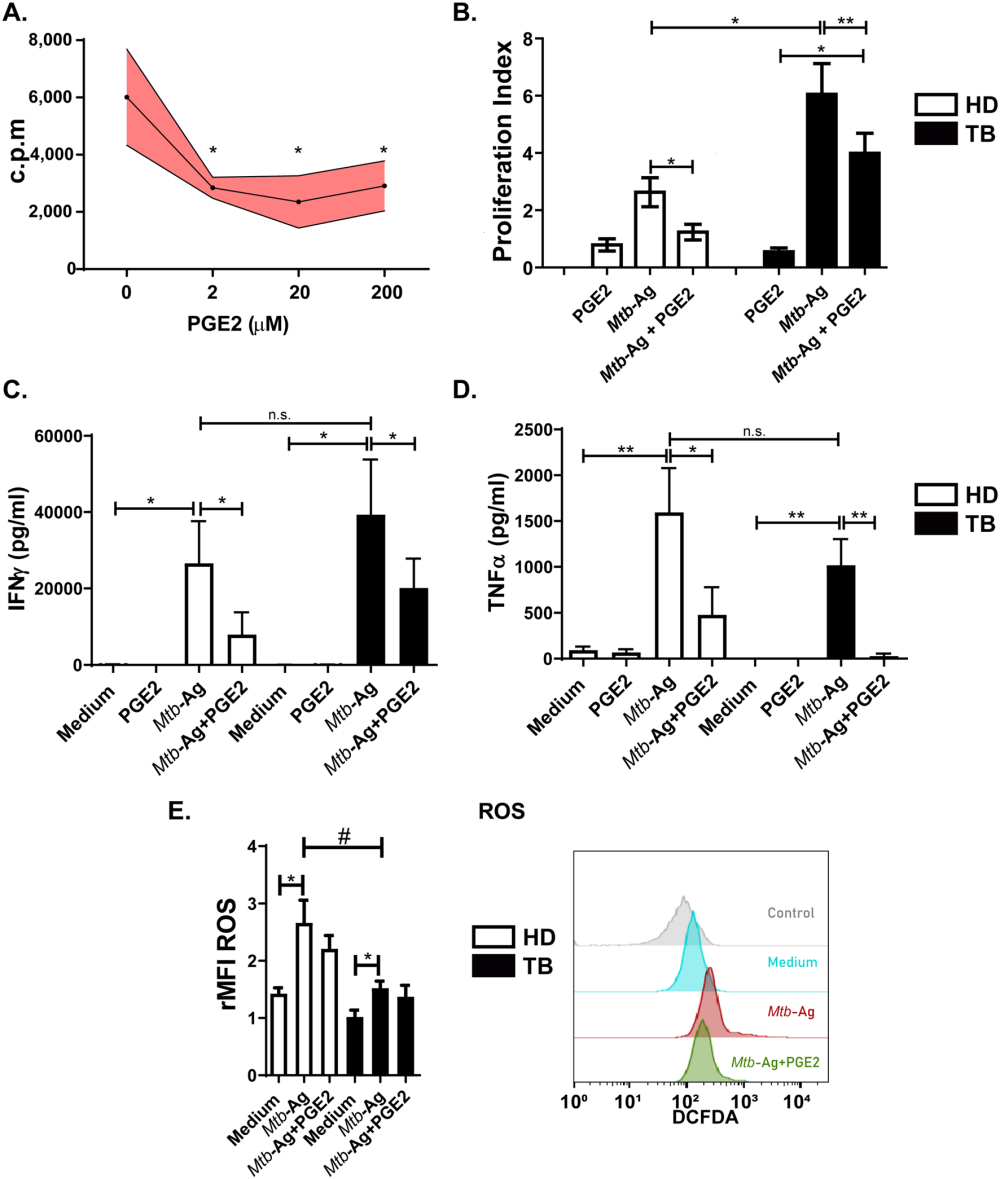
Inhibitory role of PGE2 on several functions of human immune cells. (**A,B**) PBMC from TB patients and/or HD were stimulated with *Mtb*-Ag (10 µg/ml) in the presence or absence of PGE2 for 5 days. Afterwards, cellular proliferation was analyzed by measuring the incorporation of tritiated thymidine during the last 16 h of culture. (**A**) Dose–response curve of PGE2 (0–200 µM) on proliferation of PBMC from HD (n = 7). (**B**) Effect of PGE2 (2 µM) on cell proliferation from HD (n = 7) and TB patients (n = 6). Bars show the values of the proliferation index (c.p.m. in each condition/c.p.m. media) ± SEM. (**C,D**) PBMC from HD (n = 5) and TB patients (n = 4) were stimulated with *Mtb*-Ag (10 µg/ml) in the presence or absence of PGE2 (2 µM) for (**C**) 5 days or (**D**) 24 h. Afterwards, the production of (**C**) IFNγ and (**D**) TNFα were determined by ELISA. Each bar represents the mean of cytokine production (pg/ml) ± SEM. (**E**) Neutrophils from HD (n = 5) and TB patients (n = 5) were incubated with 2ʹ,7ʹ-dichlorofluorescein diacetate (DCFDA, 50 µM) for 15 min and then stimulated with or without *Mtb*-Ag (10 µg/ml) ± PGE2 (2 µM) for 60 min. Finally, DCFDA fluorescence was evaluated to monitor ROS production by flow cytometry. Left panel: Bars represent the mean values of the mean fluorescence intensity relative to control (rMFI) of neutrophils ± SEM. Right panel: a representative histogram is shown (Control: non-stained cells). Statistical differences were calculated using one-way ANOVA and post hoc Dunnett’s multiple comparison test. * *p* < 0.05, ** *p* < 0.01.

Taking into account our findings showing a decreased expression of receptors involved in T cell activation, and to further investigate the role of PGE2 on the human immune response against *Mtb* infection, we next studied the effect of this eicosanoid on lymphocyte proliferation. Thus, PBMC from both HD and TB patients were stimulated with *Mtb*-Ag in the presence or absence of PGE2 for 5 days. Finally, cell proliferation was analyzed by tritiated thymidine incorporation over the last 16 h of culture. As shown in Fig. 3A, even at the lowest concentrations, PGE2 significantly inhibited cell proliferation induced by *Mtb*-Ag. Furthermore, a significant suppressive effect of PGE2 on cell proliferation was observed both in HD and TB patients (Fig. 3B), although in patients with active disease this was a partial effect.

Additionally, we analyzed the effect of PGE2 on the production of key cytokines that participate in the immune response of the human host against *Mtb*. Therefore, we investigated the secretion of IFNγ and TNFα from PBMC stimulated with *Mtb*-Ag in the presence or absence of exogenous PGE2. In line with the results described above, PGE2 induced a significant decrease in IFNγ and TNFα production in response to *Mtb*-Ag (Fig. 3C,D).

Furthermore, we next studied the effect of PGE2 on the intracellular levels of ROS in neutrophils from HD and TB patients. As previously reported, *Mtb*-Ag stimulation triggered ROS production^16^ (Fig. 3E). Nevertheless, although a slight decrease in ROS production was observed in neutrophils from HD, no significant differences were detected in ROS generation upon PGE2 treatment of human neutrophils (Fig. 3E).

### PGE2 promotes autophagy in *Mtb*-Ag-stimulated cells from HD and TB patients

Taken together, the findings presented so far suggested a suppressive/regulatory role for PGE2 on the innate and adaptive immune responses of the human host against *Mtb*. However, different roles for PGE2 during *Mtb* infection have been described^5^. Accordingly, PGE2 has been proposed as a host-directed treatment to counteract the predominant type I interferon response displayed by severe TB patients^6^ and to protect against cell necrosis^5,9^. In this context, autophagy arises as a potential mechanism that may explain the positive role of PGE2 during the early stages of *Mtb* infection. In this regard, it has been shown that cyclic mechanical stretching induces autophagic cell death in tenofibroblasts through the activation of PGE2 production^26^. Moreover, it has been demonstrated that nicotine induces cell stress and autophagy mediated by COX-2 activation and PGE2 production in human colon cancer cells^27^. Furthermore, it was recently described that the expression of COX-2 contributes to increase autophagy and therefore to eliminate *Mtb* in murine bone marrow-derived macrophages^15^. Nevertheless, this regulation by PGE2 has not been described in primary human cells. Therefore, we next analyzed autophagy modulation by PGE2 in *Mtb*-Ag stimulated-PBMCs from HD and TB patients.

**Figure 4:**
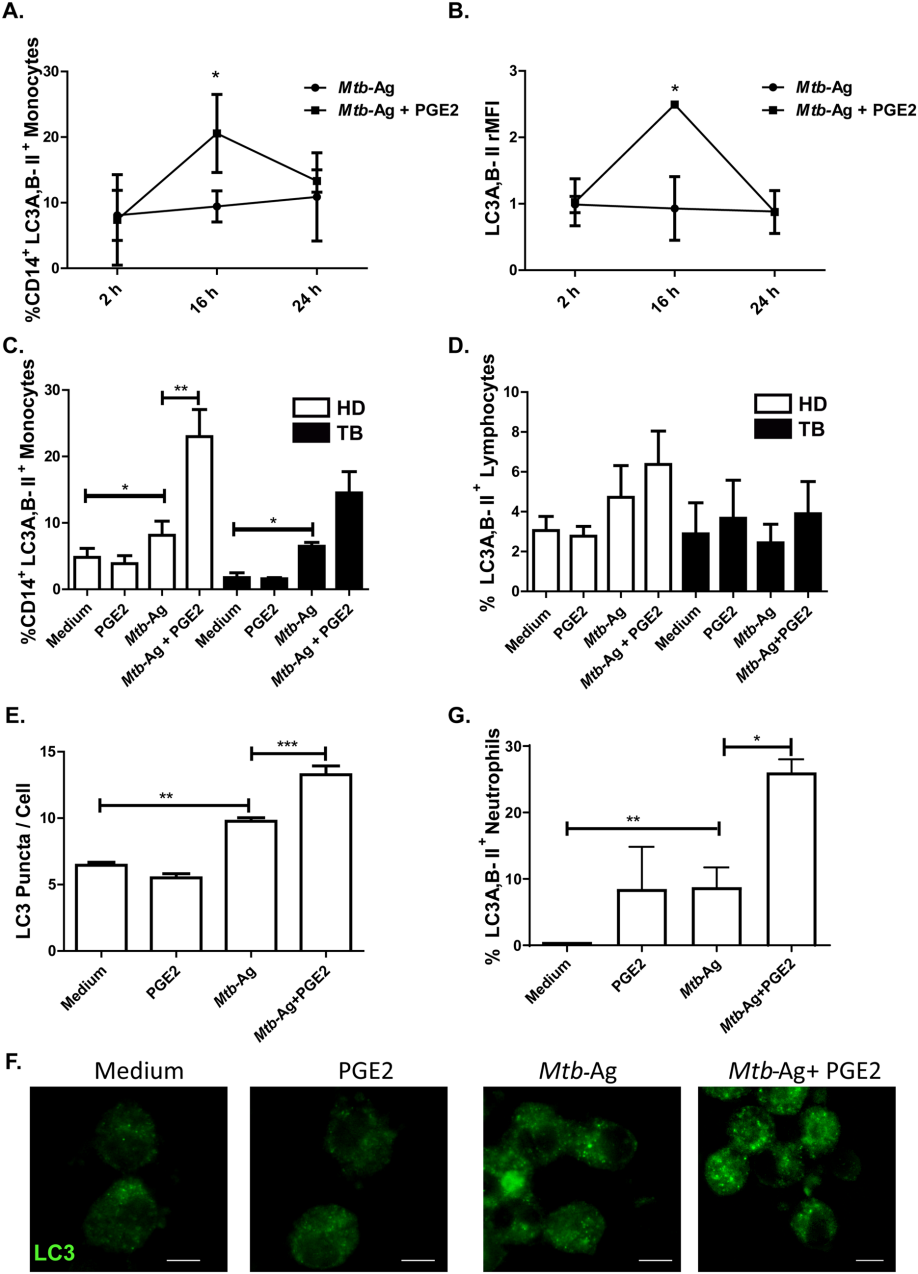
PGE2 promotes autophagy in *Mtb*-Ag-stimulated cells from HD and TB patients. PBMC from HD (n = 5) and TB patients (TB, n = 4) were stimulated with *Mtb*-Ag (10 µg/ml) in the presence or absence of PGE2 (2 µM) at different time points as indicated. The levels of autophagy were then evaluated by intracellular flow cytometry. (**A**) Percentage of CD14^+^LC3A, B-II^+^ cells ± SEM; (**B**) Relative mean fluorescence intensity (rMFI) of CD14^+^LC3A, B-II^+^ cells ± SEM. (**C, D**) Bars represent the percentage of (**C**) CD14^+^ LC3A, B-II^+^ monocytes and (**D**) CD3^+^ LC3A, B-II^+^ lymphocytes after 16 h of stimulation. (**E**) PBMC from HD (n = 4) were incubated at 2 × 10^6^ cells/ml for 16 h to allow adherence of monocytes. Afterwards, the cells were stimulated with *Mtb*-Ag (10 µg/ml) in the presence or absence of PGE2 (2 µM) for 16 h. Autophagy levels were then evaluated by immunofluorescence against LC3 on monocytes. Bars represent the mean values of LC3 puncta per cell ± SEM. (**F**) A representative experiment is shown. (**G**) Human purified neutrophils from HD (n = 7) were stimulated with *Mtb*-Ag (10 µg/ml) in the presence or absence of PGE2 (2 µM) for 2 h. The levels of autophagy were then evaluated by intracellular flow cytometry. Statistical differences were calculated using one-way ANOVA and post hoc Dunnett’s multiple comparison test. *p < 0.05, **p < 0.01, ***p < 0.001.

First, we performed a kinetic study to evaluate the autophagy levels in stimulated-cells from HD and TB patients. As shown in Fig. 4A,B, a significant increase in the percentage of CD14^+^LC3A, B-II^+^ monocytes and in the relative mean fluorescence intensity (rMFI) were detected in cells treated with PGE2 for 16 h. However, the augmented levels of autophagy markedly decreased at 24 h (Fig. 4A,B). The increased LC3A, B-II levels detected at 16 h were corroborated both in monocytes from HD and TB patients (Fig. 4C and Supplementary Fig. S9). In contrast, no significant differences in LC3A, B-II levels were observed in lymphocytes from both populations of individuals (Fig. 4D and Supplementary Fig. S9). Likewise, confocal microscopy analysis yielded similar results, denoted by a greater number of LC3 puncta per cell in *Mtb*-Ag stimulated-monocytes treated with PGE2 as compared to untreated cells (Fig. 4E,F). Interestingly, PGE2 treatment also augmented the percentage of LC3A, B-II^+^ neutrophils upon *Mtb*-Ag stimulation (Fig. 4G).

**Figure 5:**
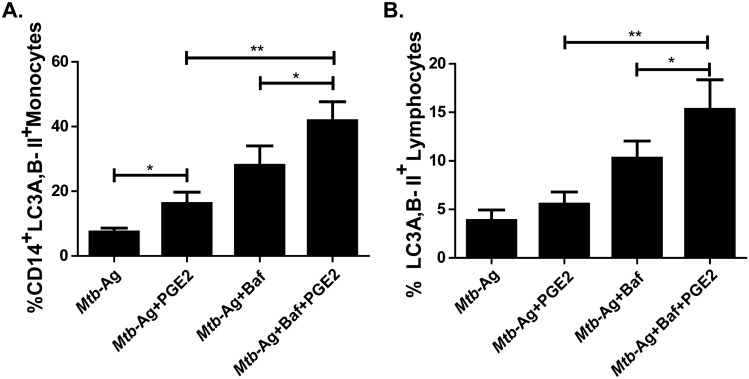
Autophagy flux in cells treated with PGE2. Peripheral blood mononuclear cells (PBMC) from tuberculosis patients (n = 6) were incubated at 2 × 10^6^ cells/ml for 16 h to allow adherence of monocytes. Afterwards, the cells were stimulated with *Mtb*-Ag (10 µg/ml) in the presence or absence of PGE2 (2 µM) for 16 h. To evaluate autophagy flow, Bafilomycin A1 (Baf A1, 1 µg/ml) was added in the last 2 h of culture before determining the autophagy levels by flow cytometry by means of indirect intracytoplasmic staining of LC3A, B-II resistant to saponin in (**A**) CD14^+^ monocytes and (**B**) lymphocytes. The bars represent the mean values of the percentage of LC3A, B-II cells ± SEM . * *p* < 0.05, ** *p* < 0.01. *P* values were calculated using one-way ANOVA and Tukey post hoc multiple comparison test.

It is important to consider that an increase in LC3-II levels can be associated both with a high synthesis of autophagosomes and with a blockage in the autophagic flow (caused by limited fusion of lysosomes or reduced activity of lysosomal enzymes) that reduces the degradation of this protein^28^. To elucidate the origin of LC3-II accumulation by PGE2 treatment^29^, cells from TB patients were stimulated with *Mtb*-Ag in the presence or absence of the eicosanoid, and incubated with Bafilomycin A1 (Baf A1) during the last 2 h of culture. In this way, we were able to corroborate that the addition of Baf A1 causes a significant increase in autophagy levels as compared to monocytes stimulated with *Mtb*-Ag plus PGE2 (Fig. 5A). This implies that PGE2 induces the formation of autophagosomes, promoting their maturation and a functional autophagic flow. Therefore, our findings demonstrate that PGE2 causes an autophagy-inducing effect in human monocytes. Interestingly, treatment with PGE2 and Baf A1 produced a significant increase in the percentage of LC3A, B-II^+^ lymphocytes as compared to cells only treated with PGE2 (Fig. 5B), corroborating an autophagy-inducing effect of PGE2 also in this cell type.

Type I IFN have been shown to suppress the production of protective host cytokines after infection with *Mtb*^30^*.* Furthermore, excessive production of type I IFN is associated with a higher susceptibility to TB^6^. Besides, several reports have described that these cytokines display an autophagy-inducing function in different pathological models such as viral infections and cancer^31,32^. However, so far, the contribution of type I IFN to autophagy modulation had not been studied in the context of human TB. Remarkably, IFNα treatment of *Mtb*-Ag stimulated-monocytes did not induce a significant increase in the percentage of CD14^+^ LC3A, B-II^+^ cells (Supplementary Fig. S10A), or in the number of LC3 foci per cell (Supplementary Fig. S10B, C). Given that IFNɑ induces NF-kB activation in different cell types^33–36^, we proved the efficiency of our IFN treatment by analyzing the subcellular localization of NF-kB in PBMC treated with and without type I IFN. As shown in Supplementary Fig. S10D, we detected activated NF-kB in the nucleus of cells cultured with IFNɑ, whereas the transcription factor was mainly observed in the cytoplasm of cells incubated with medium alone (Supplementary Fig. S10D), demonstrating the efficacy of this cytokine treatment. Interestingly, the combination of IFNɑ with PGE2 significantly augmented the levels of autophagy in *Mtb*-Ag stimulated-monocytes as measured by flow cytometry and immunofluorescence (Supplementary Fig. S10), highlighting the stimulating role of this lipid mediator in the autophagy process of human monocytes.

Overall, our findings demonstrate a potent immunosuppressive role for PGE2 during innate and adaptive human immune responses against *Mtb* infection, which could contribute to balance an excessive host response and to eliminate mycobacteria by promoting autophagy.

## Discussion

Even though TB is a curable disease with an efficient and economical anti-TB treatment, the current prolonged therapy protocols are difficult to maintain, especially in many regions of the world severely affected by *Mtb* infection^37^. The eicosanoids’ functions are not restricted to inflammatory responses and they also act as mediators of the pathogenesis process. This family of potent biologically active lipid mediators modulate both innate and adaptive immune responses in *Mtb* infection and have been suggested as possible HDT targets, but a deeper knowledge of eicosanoid dynamics during *Mtb* infection is required. HDT might provide an unexploited approach as complementary anti-TB therapies, either by increasing the ability of the host immune system to eliminate mycobacteria or by limiting collateral tissue damage associated with TB disease . In this regard, it has been suggested that manipulation of PGE2 and/or 5-LO could serve to counteract the type I IFN response in patients with severe TB as a HDT against *Mtb*^6^.

In this work, we found that TB patients exhibit significantly higher concentrations of plasma PGE2 as compared to HD (Fig. 1). Our findings are in line with other works reporting greater amounts of PGE2 in cerebrospinal fluid from meningeal TB patients as compared to control individuals^38^. Moreover, Mayer-Barber et al. also observed higher concentrations of PGE2 in plasma from patients with mild TB as compared to HD and even extended this result considering the ratio with LXA-4 production^6^. In addition, Nore et al. investigated the levels of plasma eicosanoids during different stages of *Mtb* infection (pulmonary, extra-pulmonary and latent infection), but no significant differences were detected in either PGE2 or LTB4 plasma levels between the groups at diagnosis of TB disease^39^. It is important to note that in Nore et al´s study, non-infected control individuals were included. In contrast, we investigated the role of PGE2 in patients with active TB as compared to healthy QFT negative donors. The exact contribution of each of these several lipid mediators to the immune response against *Mtb*, and the adequate balance among them during the course of infection, remains to be elucidated.

Interestingly, we observed diminished amounts of plasmatic PGE2 associated with severe clinical presentations (radiological lesions and time of disease evolution, Fig. 1B). Furthermore, a clear trend to display lower PGE2 concentration in plasma was found in TB patients with high neutrophil counts (Supplementary Fig. S4C), which was previously associated with disease severity linked to hyperinflammation^24,25^. We speculate that the anti-inflammatory properties of PGE2 could be preventing the pathology caused by an excessive immune response in this set of patients.

Given the multiple functions of PGE2 in various cell types during different stages of the immune response, this eicosanoid is paradoxically a proinflammatory factor with immunosuppressive activity. In fact, we observed that PGE2 treatment inhibited the proliferation of *Mtb-*stimulated T cells from HD and TB patients (Fig. 3). In line with our results, it has been shown that treatment with indomethacin, a selective COX-2 inhibitor, increases lymphocyte proliferation in HD and patients with TB or leprosy^40^. Strikingly, Tonby et al. reported that the use of this inhibitor decreases the proliferation and cytokine secretion of mycobacterial antigen-stimulated lymphocytes from TB patients^41^. However, these observations could be linked to a direct inhibitory effect of indomethacin on the activation of the NF-κB pathway^41^.

Optimal T cell activation requires costimulatory signaling provided by the interaction of molecules expressed on the antigen-presenting cell with specific ligands on the T lymphocyte. Therefore, we also study the effect of PGE2 on the expression of different cell receptors. We observed that 5 days of treatment with PGE2 significantly decreased the expression of SLAMF1 and CD31 on CD3^+^ lymphocytes from HD and TB patients. Accordingly, we previously demonstrated that costimulation through SLAMF1 increases IFN-γ expression in cells from TB patients^21,22^. Furthermore, we have also demonstrated that the interaction between CD31 and SAP negatively regulates the pathways that lead to IFN-γ production during human active pulmonary TB^23^. Additionally, a reduction in the surface expression of CD80 and MHC class I molecules was found on CD14^+^ mononuclear phagocytes treated with PGE2 (Fig. 2). In line with our findings, the addition of PGE2 has been shown to reduce the expression of CD46 on activated T cells^42^. Furthermore, PGE2 also inhibits the expression of CD80, CD86 and MHC class I after LPS stimulation^43^. In contrast, PGE2 might increase dendritic cell maturation in other models^44^, denoting the importance of the exposure time and PGE2 concentration, to mention a few variables.

In addition, our studies in human neutrophils revealed a decrease in the surface expression of SLAMF1 and PD-L1 after *Mtb*-Ag stimulation in the presence of PGE2 (Fig. 2). Previously, we had demonstrated that SLAMF1 promotes ROS generation during *Mtb*-Ag stimulation of human neutrophils^25^. Nevertheless, here we did not find a significant modulation of ROS production after PGE2 treatment, at least at this time point (60 min). Further investigation is needed to clarify if PGE2 affects other biological functions of neutrophils, such as migration, NET liberation, and phagocytosis, among others.

It is important to note that our data indicated that SLAMF1 and PD-L1 expression are significantly increased in *Mb*-Ag-stimulated neutrophils from HD as compared to TB patients. Furthermore, ROS production after stimulation with *Mtb*-Ag was higher in neutrophils from HD in comparison to TB patients. Accordingly, we have previously reported that TB patients displayed imbalances in the expression of the inhibitory costimulatory molecules PD-1, PD-L1, PD-L2 in T and NK cells as compared to HD^45,46^. Other authors reported elevated frequencies of PD-L1-expressing neutrophils in patients with systemic lupus erythematosus (SLE), in correlation with the activity and severity of the disease^47^. Besides, PD-L1^+^ neutrophils in tumors had been associated with disease progression and reduced gastric cancer patient survival^48^. Regarding SLAMF1, we have demonstrated that *Mtb*-Ag-stimulation induced significantly lower levels of this molecule in T lymphocytes from TB patients as compared to HD^19^. In line with our results, Bologna et al. have reported altered expression of SLAMF1 levels in cells from patients with chronic lymphocytic leukemia (CLL)^49^. Therefore, considering all these previous studies, the reduced levels of SLAMF1 and PD-L1 in neutrophils from TB patients as compared to healthy controls, might be related to an unbalance of costimulatory molecules during human pathologies. In addition, it has been recently suggested that TB disease dramatically alters neutrophil population, leading to the accumulation of heterogeneous subsets of immature and activated dysfunctional cells and a decline in true neutrophils^50^. These data suggest that a possible intrinsic defect in neutrophils from TB patients might exist. Finally, it is probable that the differential ROS production found between HD and TB patients’ cells would be related to the significant difference in SLAMF1 expression in *Mtb*-Ag stimulated neutrophils from HD and TB patients^25^.

We also observed that PGE2 treatment significantly reduced IFN-γ and TNFα secretion from *Mtb*-Ag stimulated PBMC (Fig. 3). Thus, our data indicate that PGE2 would be affecting the production of critical proinflammatory cytokines required in the immune response against this pathogen. Moreover, we analyzed the relationship between plasmatic PGE2 amounts and the immunological classification formerly reported by us^19^. Then, here we extended those results to show that LR TB patients displayed significantly higher levels of PGE2 in plasma as compared to HR TB patients (Supplementary Fig. S4A). Furthermore, patients with the lowest levels of plasma PGE2 exhibited higher proliferation and IFN-γ levels after *Mtb*-Ag stimulation as compared to TB patients with low plasmatic PGE2 amounts (data not shown). Therefore, the levels of PGE2 in patients´ plasma would be associated with both their immune response against the pathogen and their clinical state. Overall, these results suggest a clear immunosuppressive effect of elevated PGE2 levels during the human immune response against *Mtb*.

However, it remains to be understood how PGE2 might collaborate with the host response to contain *Mtb*, as suggested^6^. Protection against necrosis and the switch towards programmed cell death by apoptosis are key mechanisms in the resolution of an infection. We and other authors have demonstrated that activation of autophagy can result in the elimination of the bacteria in autolysosomes^18,51,52^. Furthermore, autophagy also controls inflammation by regulating signaling pathways of the innate immunity. In fact, autophagy removes endogenous agonists from the inflammasome and modulates the secretion of immune mediators^53^. For these reasons, we next evaluated whether PGE2 could be contributing to an autophagic response during infection with *Mtb*.

Interestingly, by using flow cytometry and confocal microscopy, we showed that treatment with PGE2 increased the levels of autophagy induced in *Mtb*-Ag stimulated PBMC. Furthermore, the exogenous addition of this eicosanoid triggered a functional autophagy flow both in monocytes and lymphocytes from HD and TB patients (Figs. 4 and 5). In different pathological models, including tenofibroblasts and human colon cancer cells, the role of PGE2 in modulating autophagy has been demonstrated^13,26,27^. Furthermore, inhibition of COX-2 has been shown to reverse autophagy induced during lupus nephritis^54^. Besides, kidney autophagy levels were shown to decrease after silencing PGES-2^55^. Importantly, Xiong et al. reported that COX-2 suppresses mycobacterial growth in murine macrophages by promoting autophagy via the AKT/mTOR pathway^15^. Strikingly, other authors described that PGE2 reverses vitamin D3-induced autophagy in macrophages infected with *Mtb*^14^*.* Nevertheless, further evaluation of autophagy flow is required in those studied cell models.

Remarkably, we detected increased autophagy in lymphocytes stimulated with mycobacterial antigens in the presence of PGE2 plus Baf A1 (Fig. 5). Numerous investigations have shown that autophagy is essential for CD4^+^ T cell survival and homeostasis in peripheral lymphoid organs. Moreover, autophagy is also required for T cell proliferation and cytokine production in response to TCR activation^56^. Besides, autophagy is activated in regulatory T cells and supports lineage stability and survival^57^. Accordingly, it has been previously demonstrated that PGE2 contributes to the expansion of regulatory T cells against mycobacterial antigens^11^. Thus, activation of autophagy by PGE2 in lymphocytes might directly participate in the preservation of these cells, and could contribute to the generalized immunosuppression observed.

In different models of viral infections, type I IFNs have been found to confer protection against these intracellular pathogens by inducing autophagy^58^. However, most studies support the findings that type I IFNs actually promote infection by *Mtb*^59,60^. Antonelli et al. have shown that type I IFN receptor deficient mice chronically infected with several *Mtb* strains showed reduced bacterial loads compared to WT infected animals^61^. Our analyses by flow cytometry and confocal microscopy revealed that human monocytes stimulated with *Mtb*-Ag in the presence of IFNα did not modify autophagy levels (Supplementary Fig. S10). However, exogenous addition of PGE2, even in the presence of elevated type I IFN concentrations, significantly induced autophagy in those cells (Supplementary Fig. S10). This finding reinforces the role of PGE2 as an inducer of monocyte autophagy, especially considering that type I IFNs promote an IFN-γ-refractory macrophage phenotype that inhibits autophagy and stimulates mycobacteria growth^62^.

Previously, some studies expressed concern about an increased risk of developing TB by taking non-steroidal anti-inflammatory drugs (NSAIDs). However, these retrospective case–control studies were associated with several limitations, especially their inability to demonstrate causality. More recently, discrepant results were obtained in murine models of experimental TB by using COX inhibitors (ibuprofen and celecoxib). The mentioned disparities were probably related to the use of different infection routes, mouse strains and timing of the experiments. These studies highlight that COX inhibition can be detrimental at the beginning of the infection but beneficial at later stages of TB disease. In this regard, Rangel Moreno et al. have described a differential participation of PGE2 during the early and late phases of experimental pulmonary TB^10^. Specifically, these authors demonstrated that COX inhibition during early infection leads to increased bacterial growth and immunopathology^10^. Thus, the inhibitory or stimulating effect of PGE2 might vary during the acute and chronic stages of TB infection, as previously suggested for other immune mediators^18,63,64^**.** Moreover, Sorgi et al. demonstrated that COX-2 inhibition significantly reduced PGE2 levels, enhanced IFN-γ production and NO release, and increased macrophage phagocytosis of *Mtb*, reinforcing the notion that optimal PGE2 levels are required for effective modulation of the immune response against *Mtb* infection^65^. Thus, timing can be key in order to advance with the use of PGE2 in the context of TB. Then, during the design of new personalized treatments focused on the regulation of the levels of host eicosanoids, various consequences will have to be considered.

Currently, different clinical trials aim to inhibit the COX-2 enzyme as a HDT during TB treatment. One of these trials employs etoricoxib together with anti-TB therapy to improve the response of the patients' immune system (NCT02503839). Another trial intends to prove the safety and efficacy of the use of ibuprofen together with anti XDR-TB treatment (NCT02781909). Furthermore, trial NCT04575519 assesses the efficacy and safety of two repurposed drugs (acetylsalicylic acid and ibuprofen) to be applied as adjunct therapy during the standard regimen for drug-sensitive and MDR TB. Finally, the NCT02060006 trial investigates the efficacy of daily self-administered Meloxicam for prevention of TB-associated Immune Reconstitution Inflammatory Syndrome (TB-IRIS) in HIV-infected adults.

In summary, our findings suggest that PGE2 displays a main suppressive role during innate and adaptive immunity against *Mtb*, participating in the homeostasis of the immune response and protecting the host from excessive inflammation. Moreover, we hypothesized that PGE2-induced autophagy could be involved in immunity suppression and bacterial clearance. Then, according to our results, a deep elucidation of PGE2 function during human TB might contribute to improve and shorten chemotherapy of TB, having a direct impact on patients´ lives. Our findings highlight the relevance of this eicosanoid during the development of a proper immune response against human TB. Thus, the role of PGE2 might particularly depend on the lenght of time that the individual has been infected with *Mtb*, the clinical status of each patient, the local concentrations of PGE2 and both the cell type and the receptor/s on which this eicosanoid is impacting. Furthermore, our results might also establish the foundations for PGE2 -based therapies, warning about the appropriate employment of NSAIDs for patients´ treatment.

## Supplementary Information

Supplementary Information.
