# Author Correction: PGE2 displays immunosuppressive effects during human active tuberculosis

**DOI:** 10.1038/s41598-021-97720-7

**Published:** 2021-09-07

**Authors:** Joaquín Miguel Pellegrini, Candela Martin, María Paula Morelli, Julieta Aylen Schander, Nancy Liliana Tateosian, Nicolás Oscar Amiano, Agustín Rolandelli, Domingo Juan Palmero, Alberto Levi, Lorena Ciallella, María Isabel Colombo, Verónica Edith García

**Affiliations:** 1 Departamento de Química Biológica, Facultad de Ciencias Exactas y Naturales, Universidad de Buenos Aires, Intendente Güiraldes 2160, Pabellón II, 4°piso, Ciudad Universitaria (C1428EGA), Buenos Aires, Argentina; 2 Instituto de Química Biológica de la Facultad de Ciencias Exactas y Naturales (IQUIBICEN), Facultad de Ciencias Exactas y Naturales, Universidad de Buenos Aires, Consejo Nacional de Investigaciones Científicas y Técnicas (CONICET), Intendente Güiraldes 2160, Pabellón II, 4°piso, Ciudad Universitaria (C1428EGA), Buenos Aires, Argentina; 3 Laboratorio de Fisiopatología de La Preñez y El Parto, Centro de Estudios Farmacológicos y Botánicos, CONICET-UBA, Buenos Aires, Argentina; 4 División Tisioneumonología,, Hospital F.J. Muñiz, Uspallata 2272, (C1282AEN), Buenos Aires, Argentina; 5 Instituto de Histología y Embriología de Mendoza, Facultad de Ciencias Médicas, Universidad Nacional de Cuyo-CONICET, CP 5500 Mendoza, Argentina

Correction to: *Scientifc Reports* 10.1038/s41598-021-92667-1, published online 30 June 2021

The original version of this Article contained an error in the spelling of the author Agustín Rolandelli which was incorrectly given as Agustín Rollandelli. The original Article and accompanying Supplementary Information file have been corrected.

