# Supplementary material for: PGE2 displays immunosuppressive effects during human active tuberculosis
Source: Sci Rep. 2021 Jun 30;11:13559. doi: 10.1038/s41598-021-92667-1 (PMC8245456; doi:10.1038/s41598-021-92667-1)
Supplement: Supplementary file 1 — Supplementary Information. [file 41598_2021_92667_MOESM1_ESM.docx]

**PGE2 displays immunosuppressive effects during human active tuberculosis**

Joaquín Miguel Pellegrini^1,2^, Candela Martin^1,2^, María Paula Morelli^1,2^, Julieta Aylen Schander^3^, Nancy Liliana Tateosian^1,2^, Nicolás Oscar Amiano^1,2^, Agustín Rolandelli^1,2^, Domingo Juan Palmero^4^, Alberto Levi^4^, Lorena Ciallella^4^, María Isabel Colombo^5^, Verónica Edith García^1,2†*^

**Supplementary Material**


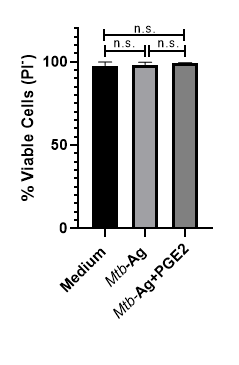


**Supplementary Figure 1.** Cell viability of treated PBMC.  PBMC were stimulated with *Mtb*-Ag in the presence or absence of PGE2 for 24 hours.  Live and dead cell populations were distinguished by flow cytometry using Propidium iodide.  n.s.: not significant. *P* values ​​were calculated using one-way ANOVA and Tukey post hoc multiple comparison test.


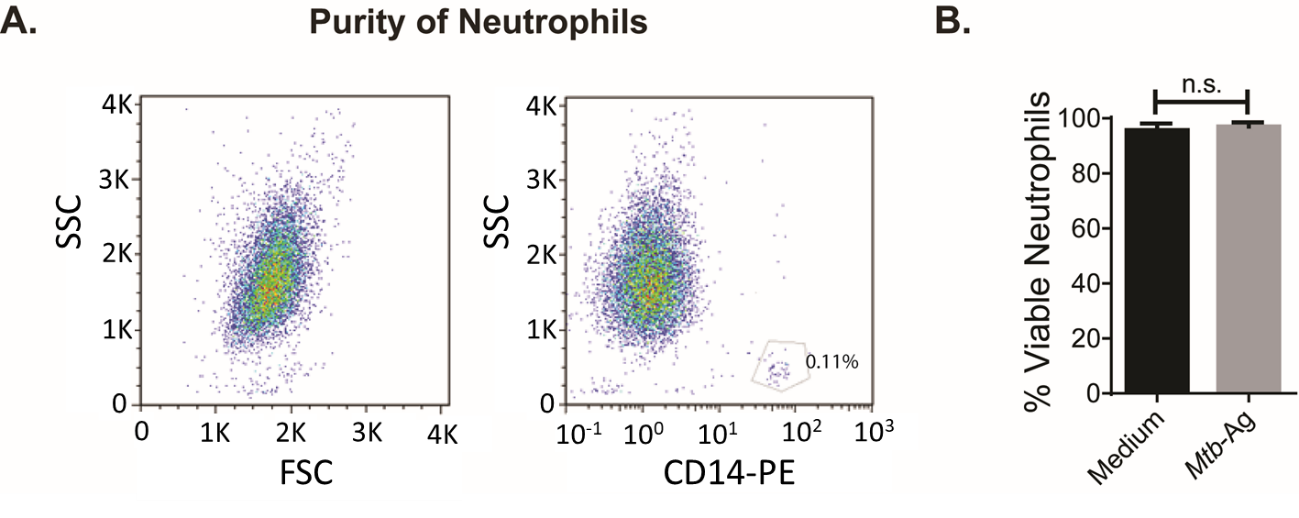


**Supplementary Figure 2.** Purity of isolated human neutrophils. Representative Dot plots of the isolated neutrophils are shown. Cells were first gated according to their size and granularity. Then, CD14 expression was analyzed to determine monocyte contamination, which was below 1%.


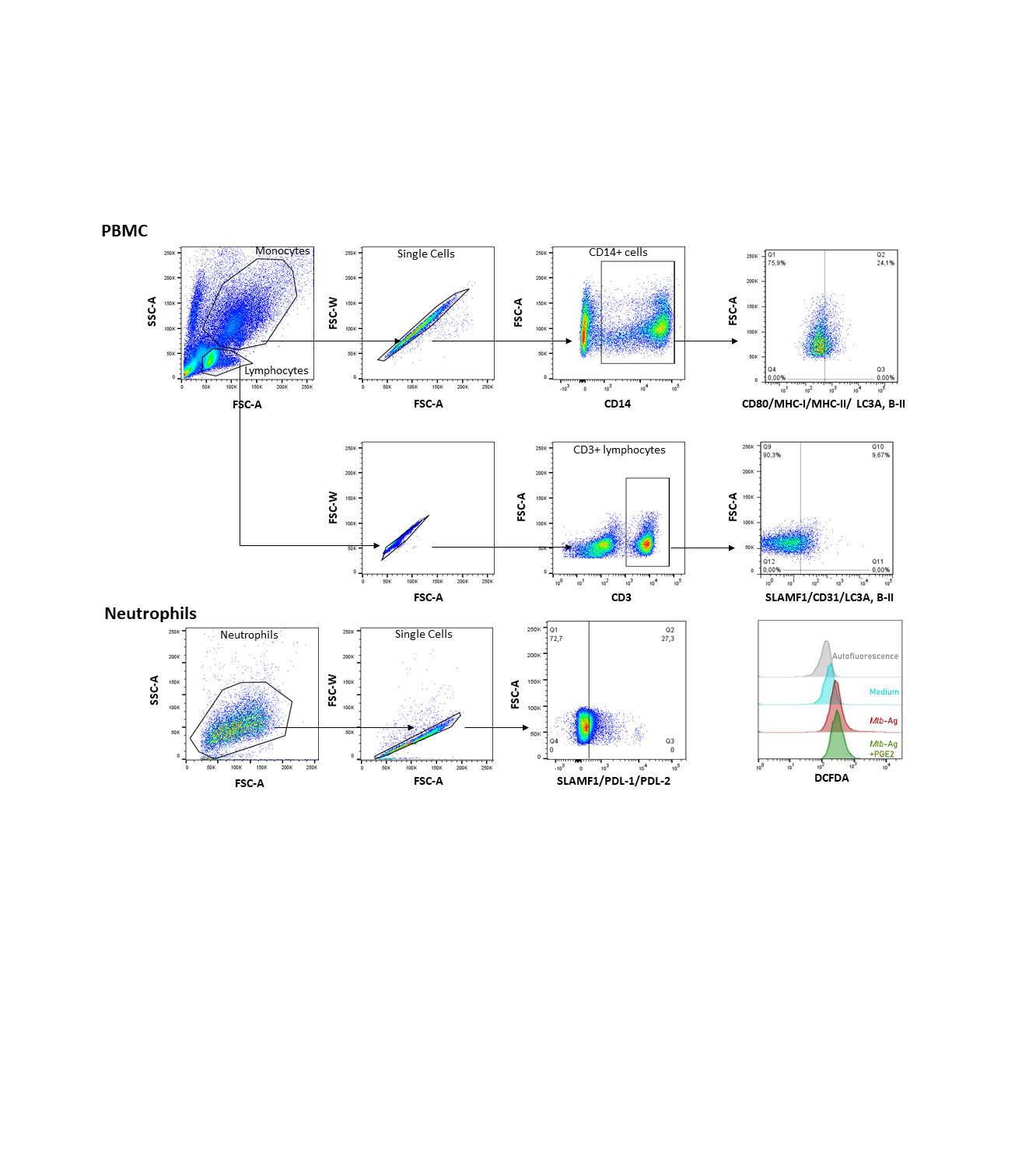


**Supplementary Figure 3.** Gating strategy for FACS analysis of PBMC and neutrophils’ receptor expression and autophagy levels. PBMC: CD3^+^ SLAMF1/CD31/LC3A,B-II^+^ T cells and CD14^+^CD80/MHC-II/MHC-II, LC3A, B-II^+^ cells were identified by flow cytometry first gating on lymphocytes or monocytes by light scatter; then, gating on singlets and on CD3^+^ or CD14^+^  cells; finally, SLAMF1/CD31/LC3A,B-II^+^ or CD80/MHC-II/MHC-II, LC3A, B-II^+^ cells were analyzed using the AND boolean gate tool from FlowJo v10 software. Neutrophils: cells were selected according to their size and granularity (light scatter) excluding debris and lymphocyte contamination (<1%) and then gating on singlets to finally analyze SLAMF1, PDL-1 and PDL-2 expression and ROS generation (DCFDA fluorescence intensity).


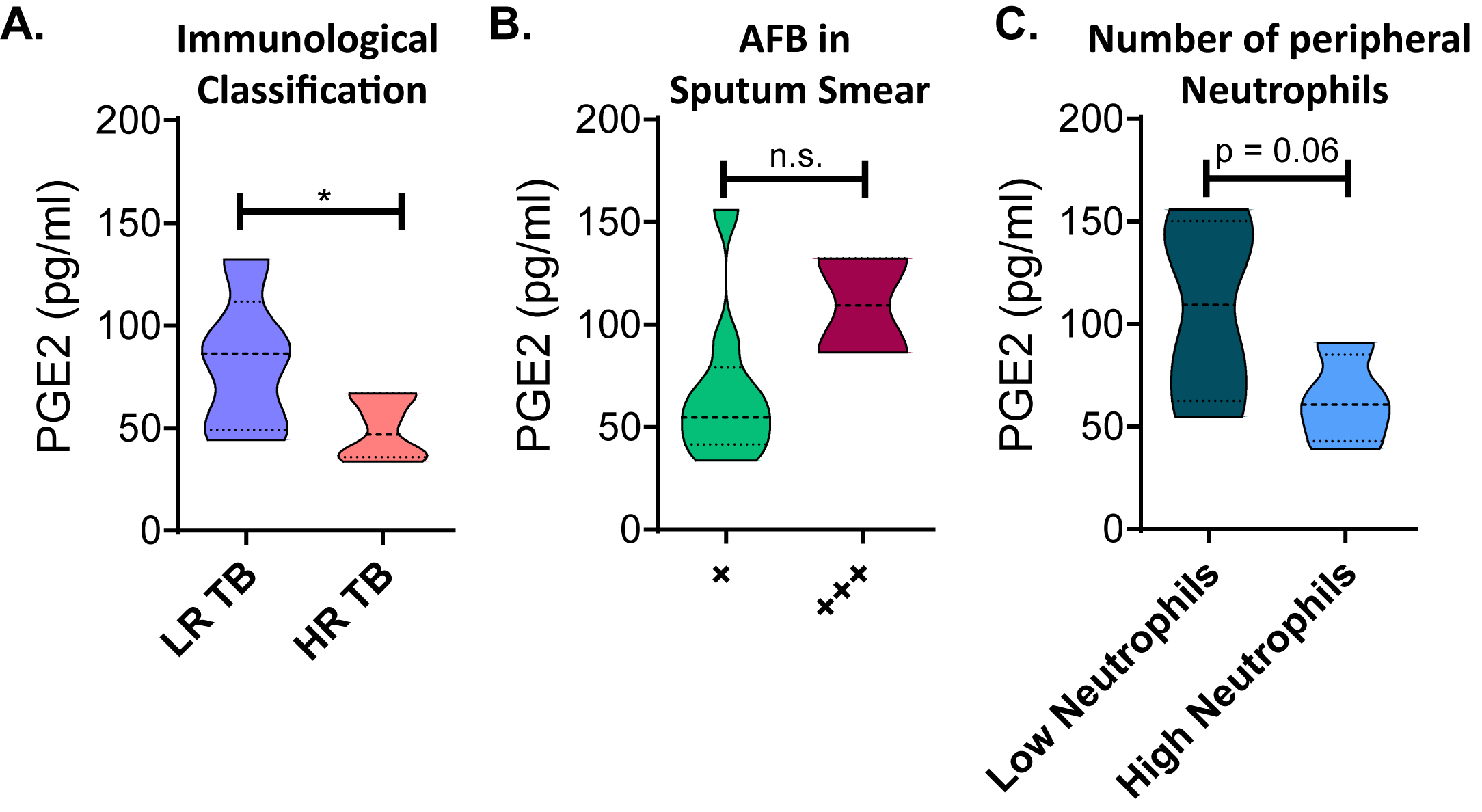


**Supplementary Figure 4.** Plasmatic PGE2 levels according to immunological and clinical classifications. Heparinized peripheral blood from TB patients (n = 13) classified according to **(A)** an immunological classification based on *in vitro* lymphocytes responses to *Mtb*-Ag (LR TB, low responder; and HR TB, high responder TB patients), **(B)** Acid-fast bacilli (AFB) in sputum smear (Ziehl-Neelsen staining); AFB +, 1–9 bacilli/100 fields; AFB +++, 1–9 bacilli/field; **(C)** neutrophil counts (cut-off determined by the median of the number of neutrophils in TB patients), was centrifuged for 15 min at 1000g and the levels of PGE2 in plasma were analyzed by RIA. Violin plots show the median values of PGE2 plasma concentration (pg/ml) ± interquartile range. P values were calculated using the Mann Whitney non-parametric test for unpaired samples, one-tailed. * p<0.05.


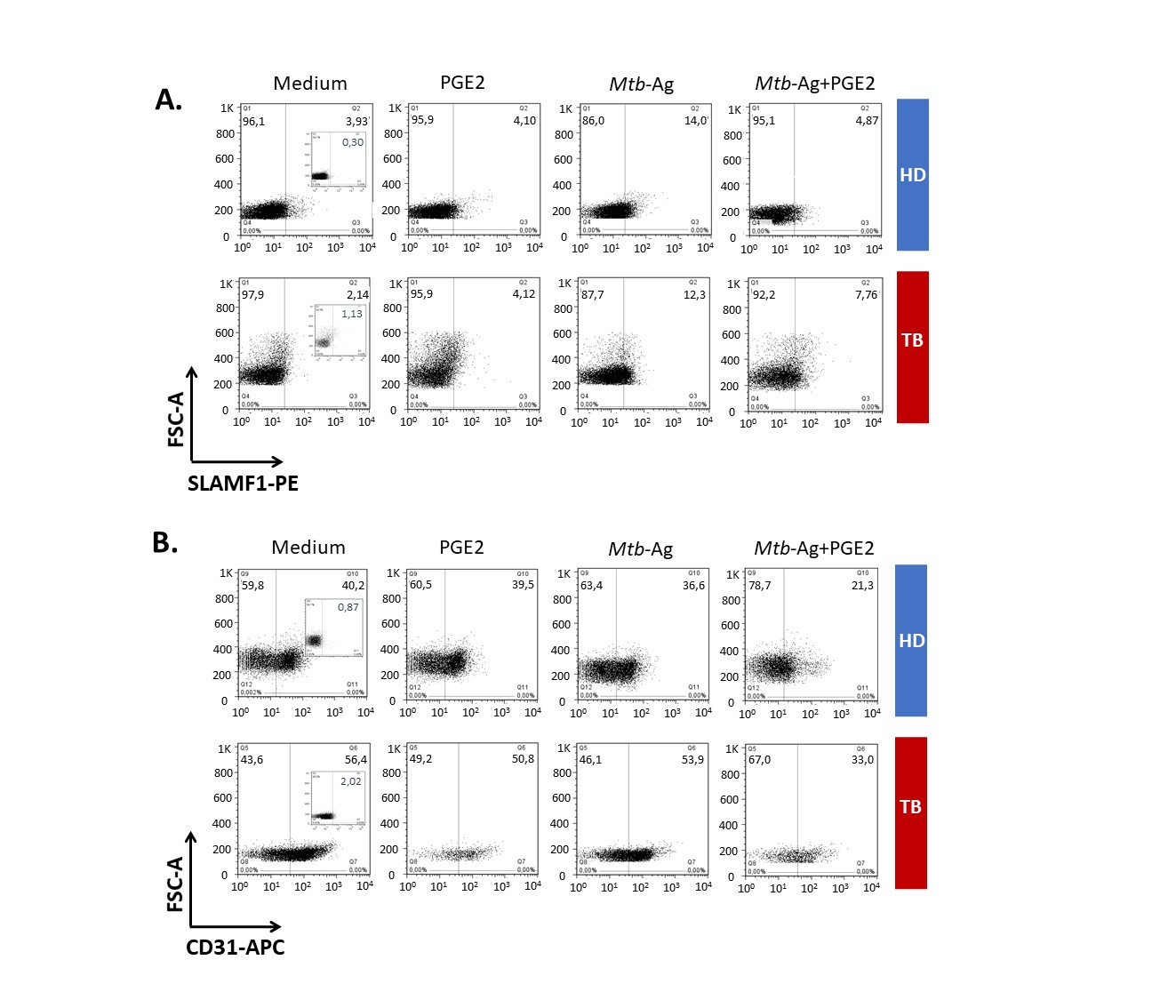


**Supplementary Figure 5.** SLAMF1 and CD31 expression on CD3^+^ cells as determined by flow cytometry. PBMC were stimulated with *Mtb*-Ag in the presence or absence of PGE2 for 5 days and the expression of SLAMF1 and CD31 on stimulated T cells were examined on CD3^+^ cells by flow cytometry. One representative HD and TB patient is shown for each group. The percentage of SLAMF1^+^ and CD31^+^ cells after culturing with media (left portion of the panel) or *Mtb-*Ag ± PGE2 (middle and right panels) (large quadrant) are shown. Isotype controls are shown in the inset.


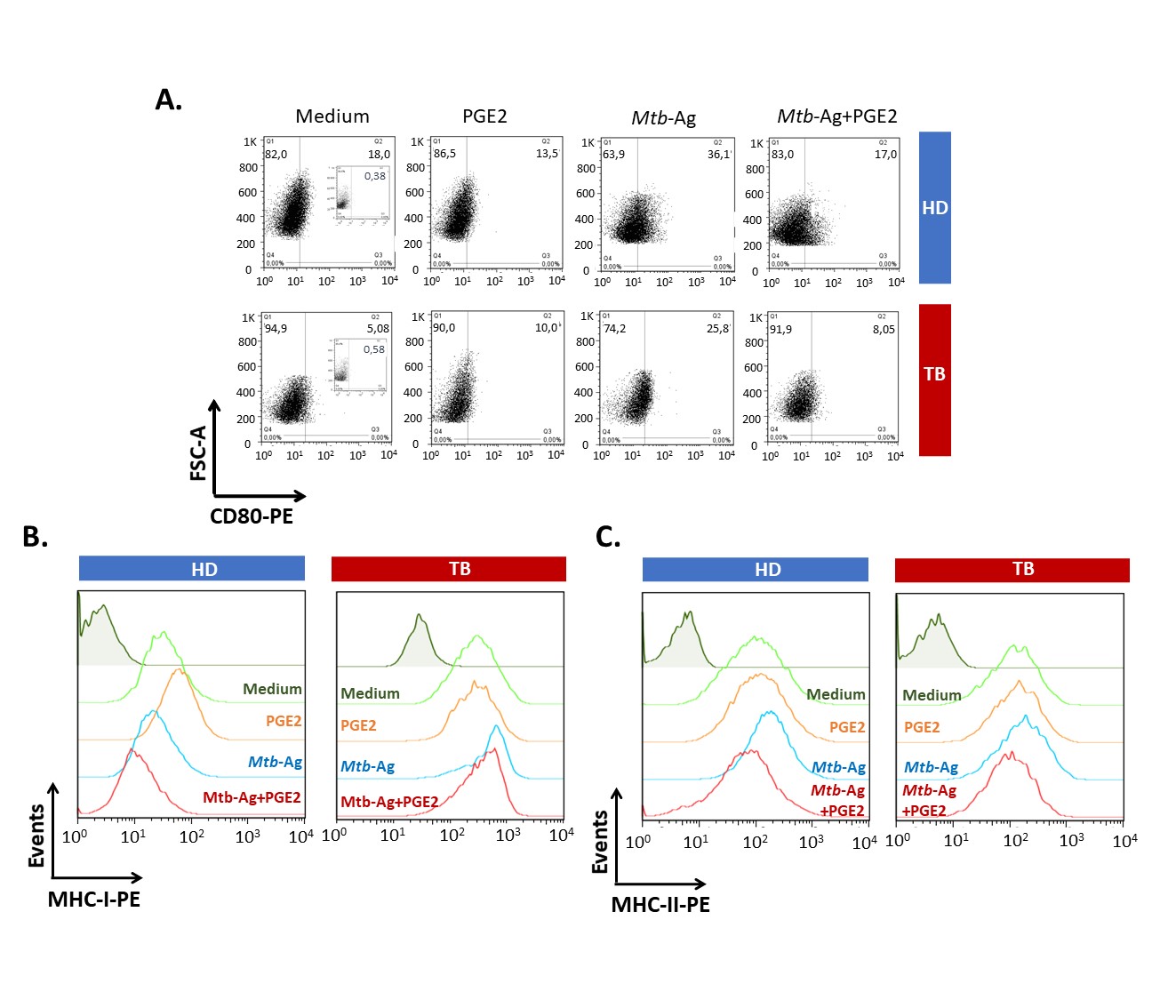


**Supplementary Figure 6.** Expressions of CD80, MHC-I and MHC-II on CD14^+^ cells from HD and TB patients were evaluated by flow cytometry. PBMC were stimulated with *Mtb-*Ag in the presence or absence of PGE2 for 5 days and the expression of CD80, MHC-I and MHC-II on stimulated cells was examined on CD14^+^ monocytes by flow cytometry.. (A) One representative HD and TB patient is shown for each group. The percentage of CD80^+^ cells after culturing with media (left portion of the panel) or *Mtb-*Ag ± PGE2 (middle and right panels) (large quadrant) are shown. Isotype controls are shown in the inset. (B) Representative histograms of flow cytometry are shown for MHC-I (left panels) and MHC-II (right panels) expression.


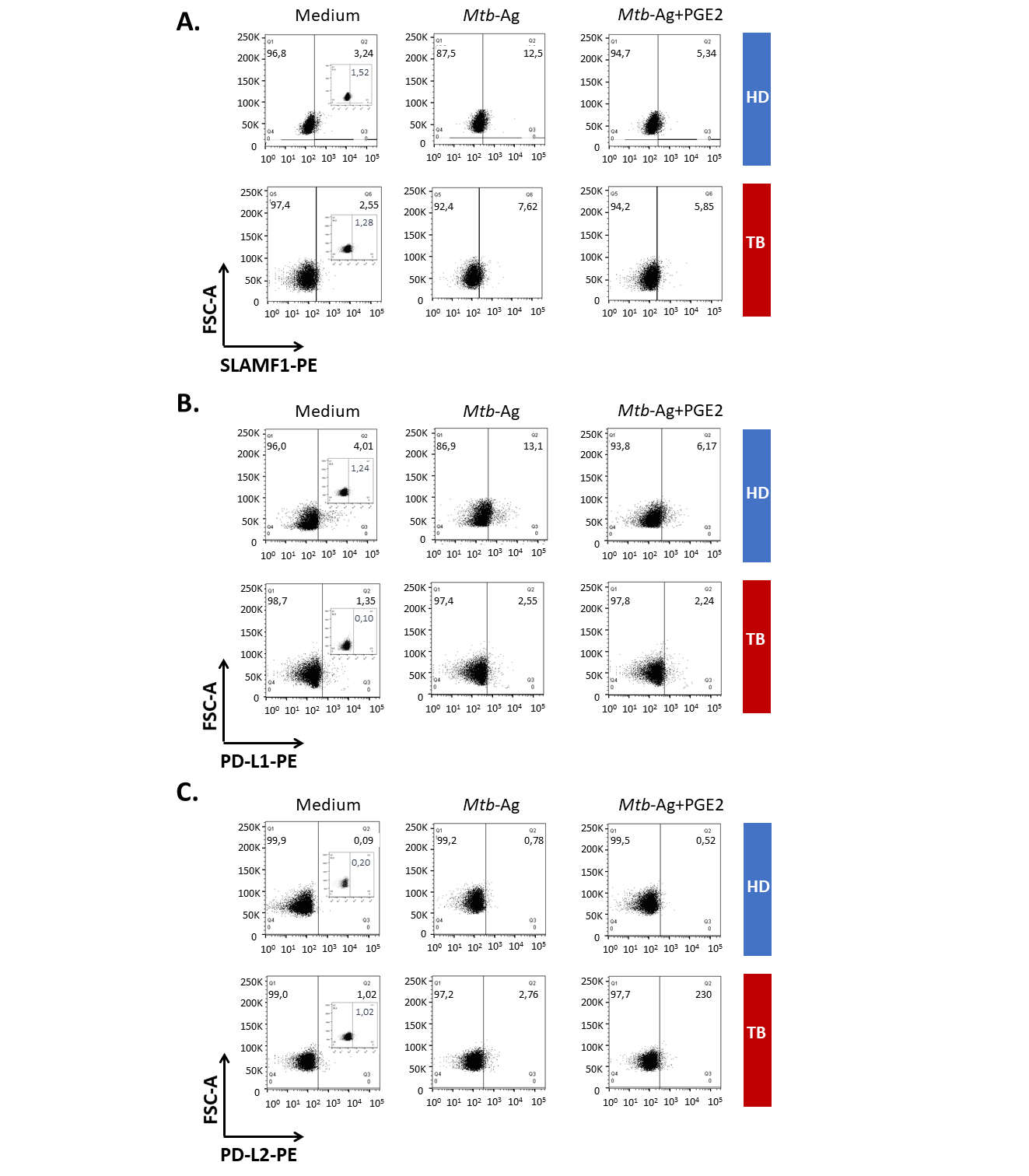


**Supplementary Figure 7.** SLAMF1, PD-L1 and PD-L2 expression on human neutrophils as determined by flow cytometry. Human purified neutrophils from HD and TB patients were stimulated with *Mtb*-Ag (10 µg/ml) in the presence or absence of PGE2 (2 µM) for 2 hours. Then **(A)** SLAMF1, **(B)** PD-L1 and (**C)** PD-L2 expression were evaluated by flow cytometry. One representative HD and TB patient of seven is shown for each group. The percentage of SLAMF1^+^ (A), (B) PD-L1^+^ and (C) PD-L2 ^+^ neutrophils after culturing with media (left portion of the panel) or *Mtb-*Ag ± PGE2 (middle and right panels) (large quadrant) are shown. Isotype controls are shown in the inset.


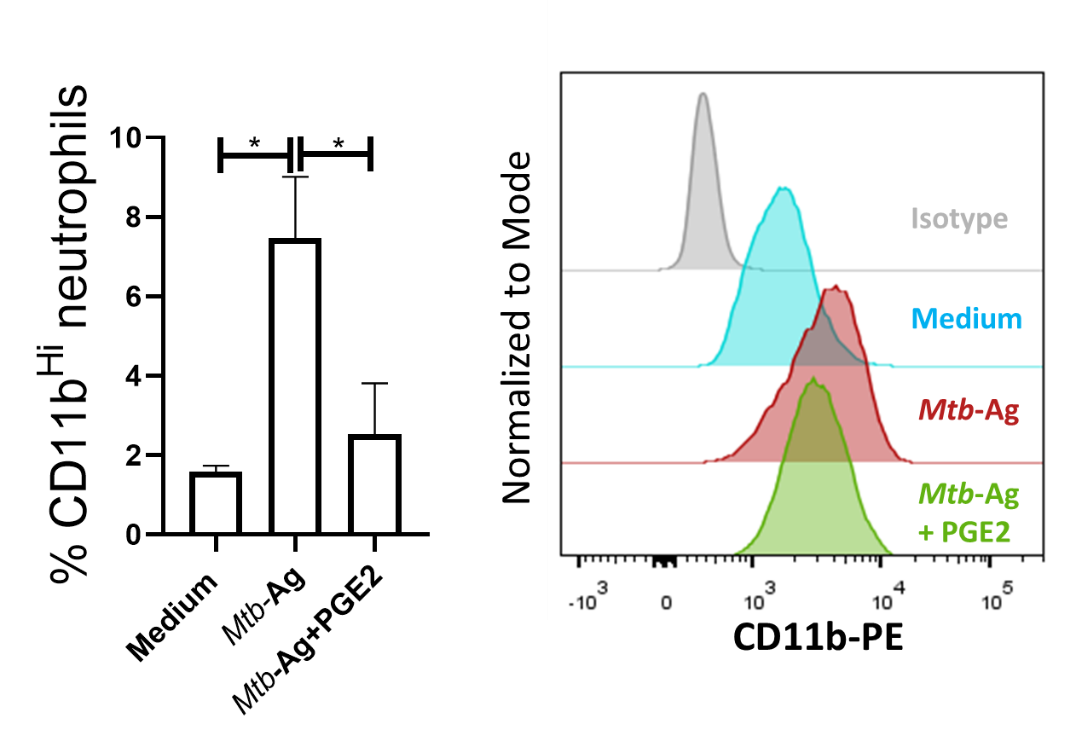


**Supplementary Figure 8. PGE2 downregulates surface CD11b expression on neutrophils from HD.** Human purified neutrophils from healthy donors (HD) were stimulated with *Mtb*-Ag (10 µg/ml) in the presence or absence of PGE2 (2 µM) for 2 hours. Finally, CD11b surface expression was evaluated by flow cytometry. Left panel: Bars represent the mean values of the percentage of CD11b^+^ neutrophils ± SEM. Right panel: a representative histogram is shown. Statistical differences were calculated using one-way ANOVA and post hoc Holm-Sidak’s multiple comparison test. * *p*<0.05


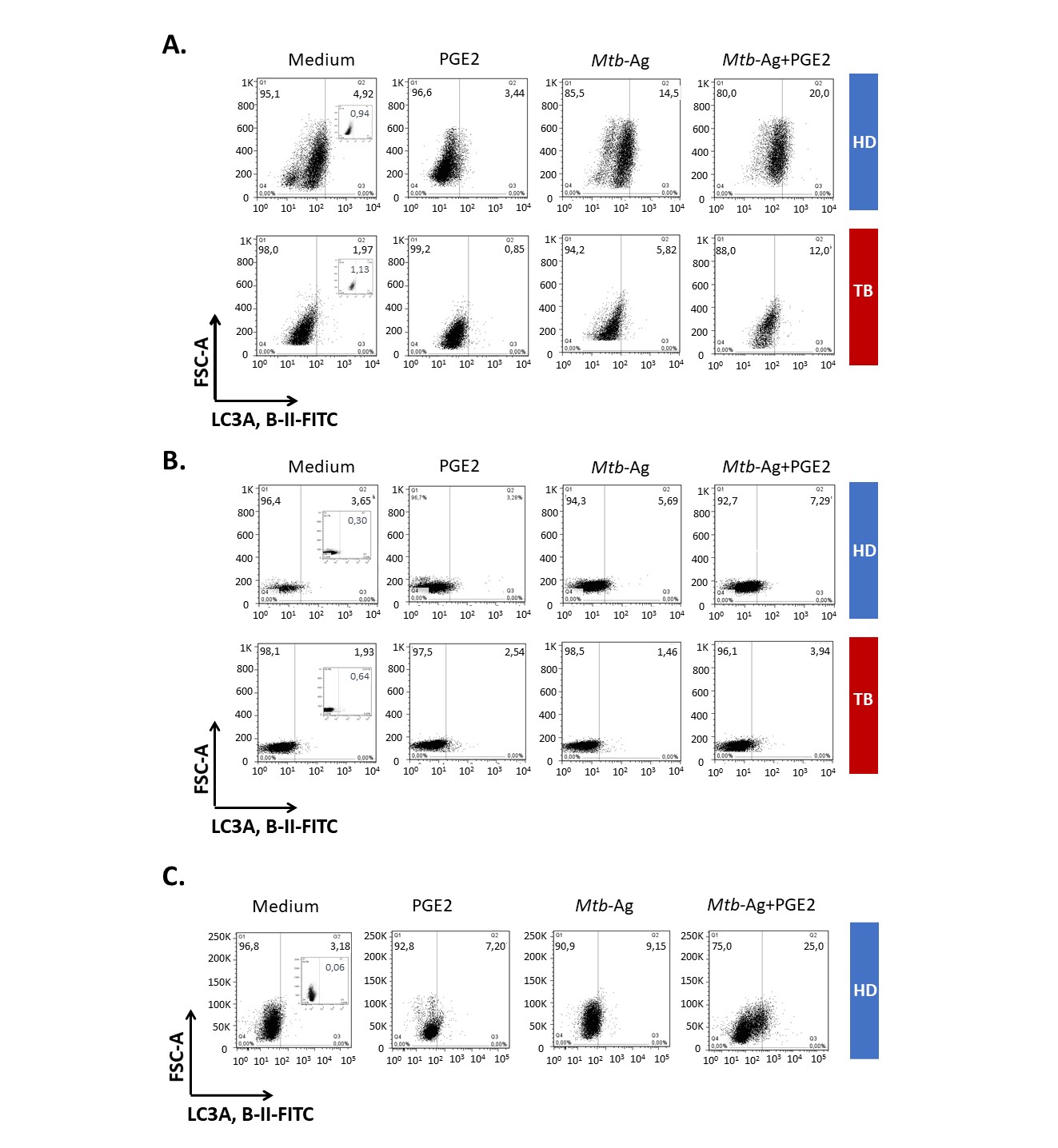


**Supplementary Figure 9.** Autophagy levels on monocytes, lymphocytes and neutrophils as determined by flow cytometry. **(A, B)** PBMC from HD (n=5) and TB patients (TB, n=4) were stimulated with *Mtb*-Ag (10 µg/ml) in the presence or absence of PGE2 (2 µM) at different time points as indicated. The levels of LC3A, B-II were then evaluated by intracellular flow cytometry in **(A)** CD14^+^ monocytes and (**B)** CD3^+^ lymphocytes after 16 hours of stimulation. One representative HD and TB patient is shown for each group. **(C)** Human purified neutrophils from HD (n=7) were stimulated with *Mtb*-Ag (10 µg/ml) in the presence or absence of PGE2 (2 µM) for 2 hours. The levels of autophagy were then evaluated by intracellular flow cytometry. One representative experiment is shown. Isotype controls are shown in the inset.

**
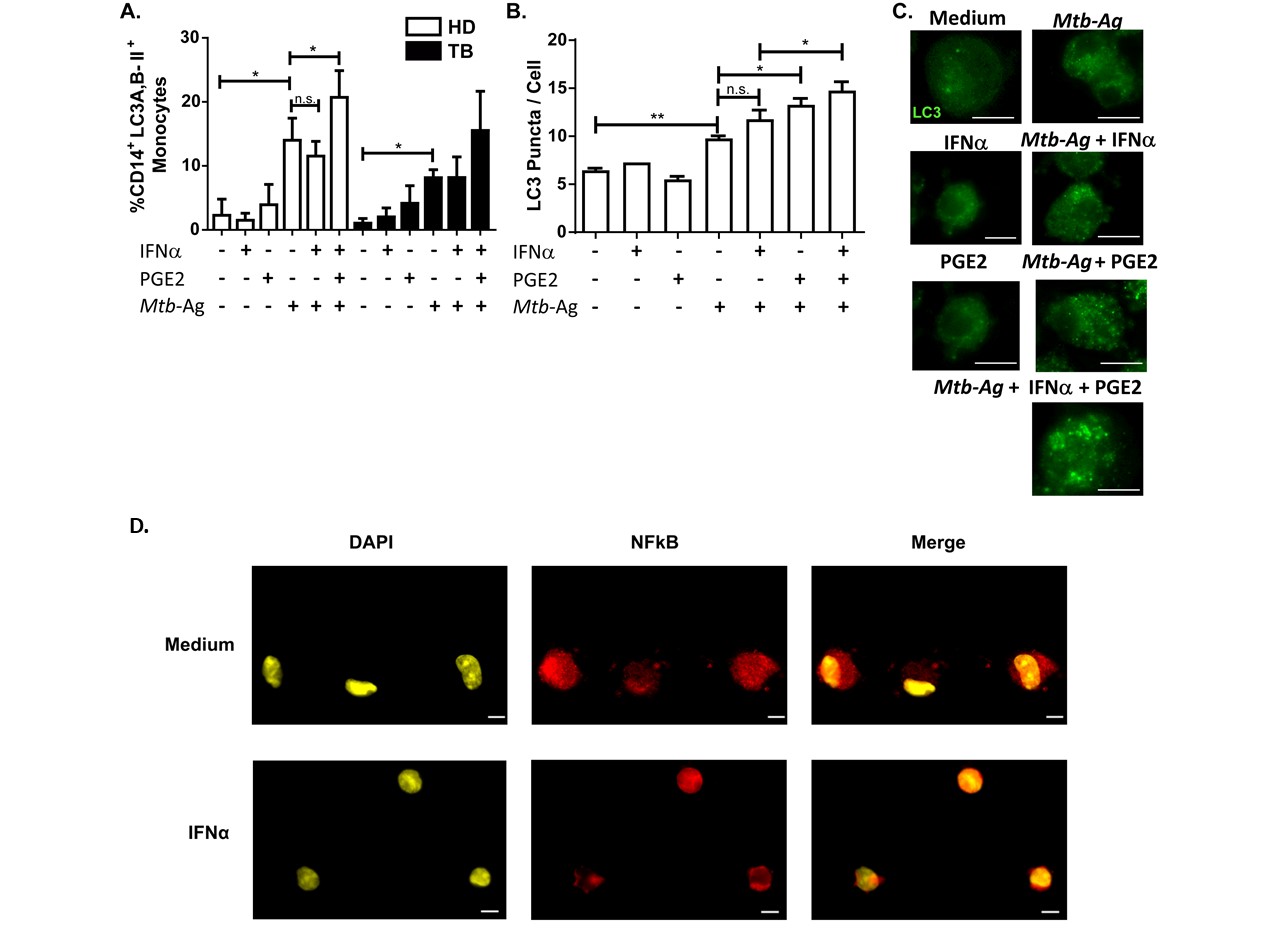
**

**Supplementary Figure 10. Effect of IFNα on the regulation of autophagy in human monocytes**. PBMC from HD (n=4) and TB patients (n=5) were incubated at 2x10^6^ cells/ml during 16h to allow adherence of monocytes. Afterwards, the cells were stimulated with *Mtb*-Ag (10 µg/ml) in the presence or absence of IFNα (10 ng/ml) with or without PGE2 (2 µM) during 16h. Autophagy levels were evaluated by **(A)** flow cytometry against intracellular saponin-resistant LC3A, B-II in CD14^+^ cells and by **(B)** immunofluorescence against LC3B in monocytes. **(C)** Representative images of one experiment are shown. Bars represent the mean values of LC3 puncta per cell ± SEM. * *p*<0.05, ** *p*<0.01. Statistical differences were calculated using one-way ANOVA and post hoc Dunnett multiple comparison test. (**D) PBMC from HD were stimulated with or without IFNɑ (10 ng/ml) for 30 minutes. Then, cells were immunolabelled with DAPI (nucleus, yellow) and anti-p65 (NFκB, red). Finally, NFkB subcellular localization was analyzed by immunofluorescence microscopy. Scale bar, 5 µm. Representative images of one experiment are shown.**
